# Virtual Reality for Information Visualization Might Just Work This Time

**DOI:** 10.3389/frobt.2019.00084

**Published:** 2019-09-04

**Authors:** Bruce H. Thomas

**Affiliations:** IVE: Australian Research Centre for Interactive and Virtual Environments, School of Information Technology and Mathematical Sciences, University of South Australia, Adelaide, SA, Australia

**Keywords:** virtual reality, information visualization, immersive analytics, head mounted displays, visual analytics

## 1. Introduction

In the 1990's, Virtual Reality (VR) was discredited as a means of presenting Information Visualization (InfoViz). Mercurio and Erickon (Mercurio et al., 1990), noted several problems required to be overcome before the potential of VR for scientific visualization could be realized: low image resolution, user navigation, small tracking volume, low tracker update rate, and poor user interfaces. Even with this negative reaction, a number of researchers continued pursuing this line of investigation (for example, the work of Bowman et al., 2003). As hardware and graphics techniques improved, plausible applicability of VR to InfoViz was slowly being realized. Currently, Immersive Analytics (Marriott et al., 2018) is an emerging direction for the use of VR and other immersive technologies to improve understanding of visualization problems.

There are a number of factors that influence my belief that VR supported InfoViz can be achieved. Firstly, the hardware is so much better today; this is regarding robustness, availability, and cost. The software support has greatly improved in recent years for VR and InfoViz. Because of these two reasons, far more people can investigate this problem today. Visualization is more advanced from the 1990s in their techniques and the problem domains they are applied to. There have been a number of successes for InfoViz. Still there are domains that are challenging for traditional visualization techniques, such as high dimensional data (Tang et al., 2016). VR is a potential tool for these challenging domains by allowing the user to visualize and interact with the data with new and different techniques (Sherman and Craig, 2018).

A major reason VR might be able to support InfoViz is the rise of commercial VR systems. These systems are now commodity devices designed for home use. As with graphics hardware, VR hardware is benefiting from being a large-scale product.

Current VR systems are complete. These systems have integrated HMDs, tracking systems, and handheld input devices (Anthes et al., 2016). Today there are well-designed input devices that are part of the commercial systems. What is important to realize is they are reliable and function with minimal set up. The computers today have much better graphics capability than ever before, and Table 1 provides an overview of the improvement of graphics engines over the decades. In comparison to previous VR systems, today's systems are inexpensive. Current systems complete systems (including computing) are approximate $(US) 3K. Five-six years ago^1^ an equivalent system would cost about $20K, and 20 years ago^2^ about $1 million.

**Table 1:** Review of graphics engine throughput.

**Graphics engine**	**Year**	**Throughput**
SGI Infinite Reality	1996	0.80 GPixels/sec
NVIDIA GeForce 8800 GTS 640	2006	10.26 GPixel/sec
NVIDIA GeForce RTX 2080 Ti	2019	118.80 GPixels/sec

Before the integrated systems of today, VR systems were assembled and created by the end user. The HMD and tracking system were independent purchases, and you had to mount the tracking sensor onto the HMD yourself. There were no mounting points on the HMD, and the HMDs all had non-standard round casings. Many users hot glued the tracking sensor to the HMD, and then you had to determine the mounted tracking sensor 6DOF position relative to the eye position. In the 1990's tracking technology was a major limiting factor for VR. The tracking volumes were the size of phone booths for many of the technologies. Additionally, you had to build your own 6DOF tracked hand controller input devices.

The requirements of the movie and games industries have driven the tools for the development of computer-generated graphics content, in particular, interactive content. As with the VR hardware, the InfoViz community can benefit from these advances driven by the mass market of the games and movie industry. There is a variety of commercially available software that is easy to use. Software such as Unity3D and the UnReal Engine supports the development of VR systems. They are compatible with the major VR consumer-grade systems, such as the HTC Vive and the Oculus range of VR systems. These games engines provide many high-level features that allow the construction of VR visualization systems. These software development system allow for cross-platform development across a number of VR, computer, and mobile platforms.

Previous to the current games engine supported VR development, everything had to be coded by hand. The systems were developed with graphics libraries such as OpenGL. There was custom higher-level support for some aspects such as scene graphs^3^, but there were no common software frameworks. The tracking systems had to be integrated with the software. As previously mentioned, the display and tracking systems were sold as separate pieces of hardware; therefore, the software could not make any assumptions about the integration of the two pieces of hardware. There was some help through abstraction libraries, such as VRPN (Taylor et al., 2001). At that time, people found it very difficult to share code.

VR systems are currently available off the shelf. Someone can now just go and buy an integrated VR system. As previously mentioned, software development environments are available that are compatible with VR systems. These systems are easy to set up and configure in less than an hour. There are large online communities to support users, and the companies providing the software development environments offer readily available system documentation and tutorials. Researchers can go out and start investigating the use of VR for InfoViz today.

## 2. Why Does Virtual Reality Help?

There are many potential reasons that may help InfoViz to be presented in a VR context. I feel these four provide strong support: presence, proprioception, engagement, and focus. Presence is the user's belief they are in the VR world; the user feels they are part of the virtual world. I like to think of the user “being in the data.” In an InfoViz context, the information becomes a surrounding environment for the user to explore.

Proprioception is one of a person's senses. It is the sense of knowing where different parts of one's part are in relation to each other. This enables a user to touch their nose when their eyes are closed. In VR, this enables users to fuse the location of their physical hand with their virtual hand (if they are collocated in ℝ^3^). This ability also allows someone to place an object to their right and understand the object is relative to their current body orientation. Mine et al. (1997) has demonstrated proprioception helps users understand the virtual world. In InfoViz this allows users to place information around them, and the users understand where the information is placed.

The process of interacting with data in VR has the potential to be engaging, Schmidt et al. (2018). Engagement covers a range of concepts when a user is operating a VR system, such as enjoyment, connectedness, and flow (Chen, 2007). Increasing a user's engagement with a visualization has the potential to increase their productivity. Engagement feeds right into the final concept of focus. VR has an unique attribute; the system removes the user's sight and hearing from the stimulus of the physical world. This isolation has the potential to provide a space to allow users to think clearly. Outside distractions can be controlled, and users can focus all their attention on the problem presented in the VR space. An exciting concept is collaboration in VR InfoViz spaces, and this is a new and ongoing research area (Cordeil et al., 2017b).

### 2.1. Interacting With the Virtual

The traditional mouse and keyboard are good for desktop UI tasks. There are decades of research optimizing these input devices. The 2D mouse was designed for 2D windows and 2D tasks such as selection, drag and drop, scrolling, and rubber banding. Keyboards are fantastic devices for symbolic manipulation, such as text and command entry. What devices are the best suited for 3D input in VR? There are well-designed input devices and graphics widgets developed for the commercial systems, but there are only a few input devices and graphics widgets designed for InfoViz in VR (Cordeil et al., 2017a).

### 2.2. Why Does VR Help in InfoViz?

VR allows users to interact with a computer application in different manners (Brooks, 1999). VR allows users to interact more naturally with the use of their hands. Leveraging the concept of proprioception, users can place information relative to their body. For example, this enables users to cluster a set of visualizations together, say to their right, while having the main set of visualizations in front of the user.

The user's ability to intuitively move their viewpoint allows for the effective use of the third dimension. When a user grabs and rotates a visualization with their hands, this is much easier to understand then employing a mouse technique for the same operation. Leveraging the user's sense of proprioception during an operation may mitigate the need for haptics. If the user understands the position the visualization relative to their body, they will naturally understand its location. Therefore, additional cues may not be required. This is not to say haptics do not play a role in immersive analytics, as data physicalization (Jansen et al., 2015) is an important area of investigation.

VR allows for new methods of interacting with data. A user can use their whole body to interact with data! A number of new interaction metaphors are envisioned for this form of interaction. The *ripping* of data is where a user grabs a selection of data and pulls the data apart to expose the common data between the selected regions. The *crushing* of data has the user grab a selection of data and compresses it into an abstraction or composite. The *tearing* of data is similar to ripping, but the data is separated along a parameter or axis of data. The *squashing* of data is crushing the data by a single parameter or axis. *Cuddling* data is the selection of data to highlight that it is significant.

These techniques are certainly different forms of interaction to those supported by a mouse and keyboard. They take advantage of the user's sense of proprioception. A downside to these techniques may be the confusing gestures for the user. While they make sense to one user, another user may have a different mental model of the techniques. The ripping and tearing methods might be too similar for the user. High energy full arm gesture can be fatiguing to a user and cause *gorilla arms* (Hincapié-Ramos et al., 2014).

## 3. ImAxes

ImAxes (Immersive Axes) (Cordeil et al., 2017a) is a recent example of employing VR as an effective visualization tool. ImAxes is an interactive multidimensional visualization tool that allows users to arrange the data axes in ℝ^3^. ImAxes is an Unity3D application and enjoys the portability of that platform. HTC Vive VR headset and controllers are the devices of choice. The room-sized tracked interaction space allows users to walk, employ their full bodies, and provides a satisfactory space for users to create galleries of data visualizations. ImAxes supports histograms, scatterplots, scatterplot matrices (SPLOM), parallel coordinate plots (PCP), and linked scatterplots. ImAxes is novel with its ability to compose high order visualizations from direct manipulation of the axes to create those visualizations.

## 4. Conclusion

In conclusion, I believe InfoViz is ready for VR, and there are recent examples supporting this claim: FiberClay (Hurter et al., 2019), BioVR (Zhang et al., 2019), and Tangible Braille Plot (Walsh et al., 2018). There are still issues to be solved for VR to be a truly useful tool. We need to increase the usability of VR applications for users. Standard UI widgets for VR need to be developed and placed in an SDK as a UI Toolkit for VR. This will allow users to have a base understanding of the usage of a VR application, such as all users know the “Open” command is in the “File” menu. Then, standard InfoViz components for VR need to be created and also placed in an SDK. This will entail the translation of InfoViz techniques into VR, but the InfoViz techniques must be optimized for use in a VR setting. At this point, new VR InfoViz techniques can be developed.

There are several key takeaways I wish to convey from these observations. Firstly, VR is ready and available for experimentation for you the reader to experiment with. Secondly, explore VR to solve InfoViz problems that are currently difficult on monitors. There are plenty of VR problems still to be remedied for InfoViz.

## Author Contributions

BT was the sole author of this paper, and contains his personal opinions. While BT acknowledges the numerous scientists who influenced his thinking, the paper is fully the work of BT.

### Conflict of Interest Statement

The author declares that the research was conducted in the absence of any commercial or financial relationships that could be construed as a potential conflict of interest.

## Footnotes

^1^Most of my comments will be for previous VR up to 2013ish.

^2^Some special comments will be for VR in the 90's when VR was discredited as a visualization display technique.

^3^http://www.openscenegraph.org/
